# Microbiota Composition during Fermentation of Broomcorn Millet Huangjiu and Their Effects on Flavor Quality

**DOI:** 10.3390/foods12142680

**Published:** 2023-07-11

**Authors:** Ke Wang, Huijun Wu, Jiaxuan Wang, Qing Ren

**Affiliations:** China Food Flavor and Nutrition Health Innovation Center, Beijing Technology and Business University, Beijing 100048, China

**Keywords:** broomcorn millet, Huangjiu, volatile flavor compounds, microorganism, correlation analysis

## Abstract

Broomcorn millet Huangjiu brewing is usually divided into primary fermentation and post-fermentation. Microbial succession is the major factor influencing the development of the typical Huangjiu flavor. Here, we report the changes in flavor substances and microbial community during the primary fermentation of broomcorn millet Huangjiu. Results indicated that a total of 161 volatile flavor compounds were measured during primary fermentation, and estragole was detected for the first time in broomcorn millet Huangjiu. A total of 82 bacteria genera were identified. *Pediococcus*, *Pantoea*, and *Weissella* were the dominant genera. *Saccharomyces* and *Rhizopus* were dominant among the 30 fungal genera. Correlation analysis showed that 102 microorganisms were involved in major flavor substance production during primary fermentation, *Lactobacillus*, *Photobacterium*, *Hyphodontia*, *Aquicella*, *Erysipelothrix*, *Idiomarina*, *Paraphaeosphaeria*, and *Sulfuritalea* were most associated with flavoring substances. Four bacteria, *Lactobacillus* (R1), *Photobacterium* (R2), *Idiomarina* (R3), and *Pediococcus* (R4), were isolated and identified from wheat Qu, which were added to wine Qu to prepare four kinds of fortified Qu (QR1, QR2, QR3, QR4). QR1 and QR2 fermentation can enhance the quality of Huangjiu. This work reveals the correlation between microorganisms and volatile flavor compounds and is beneficial for regulating the micro-ecosystem and flavor of the broomcorn millet Huangjiu.

## 1. Introduction

Huangjiu is a traditional fermented food in China with a long history of brewing and rich nutrition. Along with beer and wine, these are the three main ancient alcohols worldwide. Given its pleasant flavor, ease of use, and rich nutrition, Huangjiu is popular among consumers. The raw material in Southern Huangjiu is rice, whereas Northern Huangjiu is brewed from broomcorn millet. Broomcorn millet (*Panicum miliaceum*), native to Northern China, is one of the earliest cultivated grains and the most important food crop in the Yellow River [1]. The seeds are starchy and suitable for porridge and cookies. Broomcorn millet has a higher nutritional value than wheat and rice and is rich in protein, vitamins, zinc, copper, manganese, and other nutrients. Broomcorn millet is widely used as a raw material for Huangjiu brewing in China [1].

Flavor is a major sensory property affecting Huangjiu’s quality perception and is determined by diverse volatile flavor compounds [2]. Therefore, various high-precision instruments were used to detect and analyze Huangjiu’s volatile flavor compounds. Gas chromatography olfactometry (GC-O) identified 66 flavor compounds, of which 21 odorant active substances were regarded as the main aroma components in millet Huangjiu [3]. A total of 267 different volatile flavor compounds were detected and measured in Huangjiu fermented from corn using GC×GC-TOF-MS methods [4]. Analysis of the flavor volatiles revealed 64 compounds in the representative Shaoxing Huangjiu, among which esters were the most abundant [5]. At present, many studies on Southern Huangjiu have been reported, mainly including finished wine. Therefore, studies on flavor substances in the Northern Huangjiu fermentation mash have rarely been reported.

Huangjiu’s open fermentation environment can produce diverse microorganisms during the brewing process. The flavor components of Huangjiu are closely related to various microorganisms. Microorganisms play an important role in the formation of the unique flavor of Huangjiu [2]. According to Yang et al., *Weissella* and *Enterobacter* contribute greatly to the alcohol, ester, and aldehyde accumulation during Huangjiu brewing [6]. Several studies have demonstrated that some lactic acid bacteria (LAB) are the main factors in amino acid and lipid hydrolysis [7,8]. *Lactobacillus* and *Bacillus* play important roles in the formation of flavor substances [9]. Yan et al. analyzed the top five dominant genera of flavor-producing microbes, and the content of malic acid and citric acid gradually improved through millet Huangjiu fermentation [10]. Huang et al. found that *Gluconacetobacter*, *Lactobacillus*, *Lactococcus*, *Pichia*, *Wickerhamomyces*, and *Saccharomyces* were the core functional microbiota responsible for the production of the main volatile compounds in Wuyi Hong Qu glutinous rice wine [11]. Another study showed that Qu has significant differences in microorganism structure in different regions [12]. The volatile compositions of different Qu-based Huangjiu samples have almost no similarities [11]. Thus, changes in the raw materials directly influence the composition of microbial communities and alter the flavor compounds in Huangjiu.

Northern Huangjiu brewing is usually divided into two stages: primary fermentation and post-fermentation. The primary fermentation temperature is 30 °C for 5–7 days, which contributes to the growth of microbes, and it is the main stage of flavor substance formation. The post-fermentation temperature is 15 °C for 30–60 days; the low temperatures increase the accumulation of aroma compounds. Volatile aroma components are the main determinants of the style and quality of Huangjiu. Understanding the nature of the microbial communities involved in millet Huangjiu’s primary fermentation processes is of great significance for improving the flavor quality of millet Huangjiu products [13]. 

In this study, we aimed to explore microbial diversity and analyze the relationship between volatile compounds and dynamic microbial communities in broomcorn millet Huangjiu’s primary fermentation. This study determined the volatile flavor compounds in broomcorn millet Huangjiu by HS-SPME/GC–MS. Moreover, 16S rRNA and ITS rRNA gene clone libraries were used to describe bacterial and fungal diversity. The relationships between bacterial or fungal genera and flavor compounds were predicted using the Pearson correlation coefficient (r) correlation network.

## 2. Materials and Methods

### 2.1. Materials

Broomcorn millet was collected from Zhangjiakou (Hebei Province, China). A Gel Extraction Kit was purchased from Qiagen (Qiagen, Germany). The TruSeq DNA PCR-Free Sample Preparation Kit was obtained from Illumina (San Diego, CA, USA). All other reagents were purchased from Sigma-Aldrich (Saint Louis, MO, USA).

### 2.2. Sample Collection

Broomcorn millet was collected from Zhangjiakou, Hebei Province, China. The primary fermentation was carried out in the winery’s experimental fermentation tank at 30 °C for 6 days. Broomcorn millet was washed and cooked for 30 min before fermentation. Wheat Qu was added according to conventional winery standards. Presaccharification was performed before fermentation using the saccharifying enzyme. Fermented mash (approximately 300 g) was sampled on days 1, 2, 3, 4, 5, and 6 of fermentation. Three parallel samples were prepared. The collected samples were sealed in sterile plastic bags and stored at −20 °C before further analysis.

### 2.3. Physiochemical Characteristics and Volatile Compounds during Brewing

The reduced sugar content was detected using the dinitrosalicylic acid method (DNS) with glucose as the standard substance [14]. According to the GB/T13662-2008 standard, the degree of alcohol consumption and acidity was measured to evaluate the quality of Huangjiu.

The volatile flavor compounds were measured using HS-SPME-GC-MS. Each Huangjiu sample (8 mL) was mixed with NaCl (2.5 g NaCl and 5 mL 65.76 mg/L 2-octanol) in a 15 mL SPME glass vial. For extraction, the vials were incubated in a water bath and ultrasonicated for 45 min at 50 °C and then extracted using SPME fibers coated with 50/30 μm DVB/CAR/PDMS (Superco, Bellefonte, PA, USA). The flavor compounds in the samples were analyzed by QP2010 Plus-GC MS (Shimadzua, Kyoto, Japan). High-purity helium was used as the carrier gas at a constant flow rate of 1.0 mL/min, and the split ratio was set to 50/1. The temperature program was set to 35 °C for 4 min, increased to 150 °C at 5 °C/min, held at 150 °C for 2 min, then followed by a second increase to 210 °C at 3 °C/min. The temperatures of the detector and injector were both 230 °C, and that of the ion source was 200 °C. The ion energy for the electron impact (EI) ionization source at a fragment voltage of 70 eV with a scanning range of m/z 30–350. The relative content of each volatile compound was counted according to the area normalization method [15].

### 2.4. DNA Extraction and High-Throughput Sequencing

Total genomic DNA from fermented mash samples was extracted using the cetyl trimethylammonium bromide (CTAB) method, which was further adjusted to a uniform final concentration of 1 ng/μL using sterile water [16].

Hypervariable regions of V3-V4 on the 16SrRNA gene of bacteria were amplified by PCR with specific primers 338F (5′-ACTCCTACGGGAGGCAGCAG-3′) and 806R (5′-GGACTACHVGGGTWTCTAAT-3′). For fungal analysis, the internal transcribed spacer (ITS) regions of the rRNA were amplified using ITS1 (5′-Axxx CTTGGTCATTTAGAGGAAGTAA-3′) and ITS2 (5′-BGCTGCGTTCTTCATCGATGC-3′). All PCR reactions were performed using the Phusion High-Fidelity PCR Master Mix (New England Biolabs, Ipswich, Massachusetts, Country). The PCR products were detected on agarose gels and further purified using a Gel Extraction Kit (Qiagen, Germany). A sequencing library was constructed using the TruSeq DNA PCR-Free Sample Preparation Kit (Illumina, San Diego, CA, USA) following the manufacturer’s recommendations. Library quality was assessed using the Agilent Bioanalyzer 2100 system (Agilent, Santa Clara, CA, USA). Finally, the library was sequenced using an Illumina HiSeq2500 platform.

Paired-end reads were assigned to samples based on their unique barcodes and truncated by cutting off the primer sequences and barcodes. Paired-end sequencing reads were merged using FLASH (V1.2.7) [17]. According to QIIME (V1.7.0), the remaining high-quality clean data were selected for analysis after the raw reads were filtered under specific filtering conditions [18,19]. The UCHIME algorithm was used to detect chimeric sequences using the reference database (Gold database) and then removed [20,21].

### 2.5. Operational Taxonomic Units (OTU) Cluster, Species Annotation, and Phylogenetic Analysis

Sequences were analyzed using the Uparse software (Uparse v7.0.1001) [22]. Sequences with ≥97% similarity were assigned to the same OTUs. Based on the RDP3 classifier (Version 2.2), the GreenGene Database was used to annotate the taxonomic information for each representative OTU [23,24]. Both alpha and beta diversity analyses were performed based on standardized data. MUSCLE (version 3.8.31) [25] investigated the phylogenetic relationships of different OTUs using multiple sequence alignment. Alpha diversity was analyzed using six indices: observed species, Chao1, Shannon, Simpson, ACE, and coverage. All indices were calculated using QIIME (Version 1.7.0) and displayed using R software (Version 2.15.3). Beta diversity was calculated using weighted and unweighted UniFrac with QIIME software (v1.7.0).

### 2.6. Correlations between Microorganisms and Flavor Compounds

Correlations between microbial communities and volatile flavor compounds were established using the Pearson correlation coefficient (r) during broomcorn millet Huangjiu fermentation. The R programming language was used to create the correlation matrix. The *p*-value was adjusted by FDR based on the Benjamini–Hochberg method. Statistical significance was set at *p* < 0.05.

### 2.7. Preparation of Wheat Qu and Fortified Qu for Huangjiu

Wheat Qu was produced by natural fermentation for 30 days. Three bacteria from isolated wheat Qu were identified as R1 (*Lactobacillus*), R2 (*Photobacterium*), and R3 (*Idiomarina*), respectively, which were associated with flavoring substances, and a dominant bacterial strain (R4) belongs to the *Pediococcus*. After that, 1 kg of broomcorn millet was cooked with 3 kg of water. After adding 0.15% saccharifying enzyme at 60 °C for 30 min, R1, R2, R3, and R4 were incubated at 37 °C for 48 h, during which they were stirred several times. The fortified Qu was prepared as follows. The wheat was crushed, 20~30% water and 1% R1, R2, R3, and R4 strain expansion solution was added, then stirred, stepped, and stacked, and fermented naturally at 45–50 °C for 30 days to prepare fortified Qu QR1, QR2, QR3, and QR4.

### 2.8. Huangjiu Brewing and Measured Indices

Huangjiu was brewed with fortified qu. Briefly, after broomcorn millet cooking, when the temperature of the broomcorn millet rice had decreased to 60 °C, activated saccharifying enzyme was added for 30 min, and added 16% fortified qu and 0.15% yeast for 5–8 days of primary fermentation at 30 °C, followed by post-fermentation at 13–17 °C for about 1 month. After fermentation, the wine was filtered and sterilized through vacuum filtration and preserved for 2–3 days. The bottom layer of turbidity was removed to obtain Huangjiu, which was finally autoclaved at 85 °C for 30 min.

The content of biogenic amines was detected using HPLC (Agilent, Santa Clara, CA, USA) according to GB 5009.208-2016. The volatile flavor substances in Huangjiu were quantified as described before [26]. The amino acid nitrogen, non-sugar solid content, total sugars, alcohol, and acidity of Huangjiu were measured using standard methods (GB/T 13662-2018). All experiments were performed in triplicate (*n* = 3).

### 2.9. Statistical Analysis

Each experiment was carried out three times. All results are expressed as mean values ± standard deviation. SPSS 22.0, Origin 8.0, and Microsoft Office Excel 2019 were used to process the microbial and GC-MS data.

## 3. Results

### 3.1. Changes of the Acid, Reducing Sugar, and Alcohol during Broomcorn Millet Huangjiu Primary Fermentation

Physicochemical parameters, including acid, reducing sugar, and alcohol, were measured during broomcorn millet Huangjiu’s primary fermentation. As shown in Table 1, the acid contents of fermented samples all increased during primary fermentation, especially from the 3rd day to the 4th day, which ranged from 2.71 g/L to 4.90 g/L. The alcohol concentration tended to increase rapidly at the start of fermentation. The reduced sugar reached its peak value on the 2nd day (32.35 g/L) and decreased gradually with the fermentation process.

### 3.2. Changes in Volatile Flavor Compounds during Broomcorn Millet Huangjiu’s Primary Fermentation

A total of 161 volatile flavor compounds, including 43 alkanes, 33 alcohols, 31 esters, 22 ketones, 15 aldehydes, 7 acids, 3 phenols, and 7 other kinds of volatile compounds of fermented broomcorn millet Huangjiu samples, were measured during primary fermentation (see Supplementary Table S1). At the end of primary fermentation, 70 flavor compounds were identified, including 5 alkanes, 16 alcohols, 19 esters, 13 ketones, 9 aldehydes, 3 acids, 1 phenol, and 4 other compounds. Among these compounds, alcohols were the main volatile aroma component in the broomcorn millet Huangjiu with a content of 42.45% on the last day. The number of alcohols showed a growth pattern from day 1 to day 3 of fermentation and then slightly decreased. In primary fermentation, the major alcohols from broomcorn millet Huangjiu were 2,3-butanediol, glycerol, 2-phenylethanol, and 3-methyl-1-butanol. 2-phenylethanol, with a unique rose aroma, gives Huangjiu a pleasurable flavor. Furthermore, the number of esters was the largest volatile compound in the samples, which ranged from 4 to 19. The ester-relative content showed an increasing tendency from day 1 to day 5 of fermentation (18.41%) and then decreased on the last day (4.66%), as identified in this study. Among these 33 esters, ethyl caproate, hexadecanoic acid ethyl ester, 9,12-octadecadienoic acid ethyl ester, and ethyl oleate were the principal constituents.

Additionally, the acids included butyric, propionic, acetic, and caproic acids, which greatly influence flavor and Huangjiu aging [27]. Seven acids were detected in primary fermentation samples. Two acids, acetic acid and hexanoic acid, can be formed as major acids. Key substances identified among the aldehydes included glycolaldehyde, 5-hydroxymethylfurfural, furfural, and 5-methyl-2-Furancarboxaldehyde. The relative contents of alkanes decreased rapidly from day 2 to day 5 (17.49%) and increased on the last day (29.86%). The number of alkanes in the Huangjiu decreased significantly to 5. Ketones showed a decreasing tendency during brewing. Aldehydes, phenols, and other compounds exhibited slight variation.

### 3.3. Bacterial Diversity of Broomcorn Millet Huangjiu during Primary Fermentation

#### 3.3.1. Analysis of Bacterial Community Dynamics

**Figure 1:**
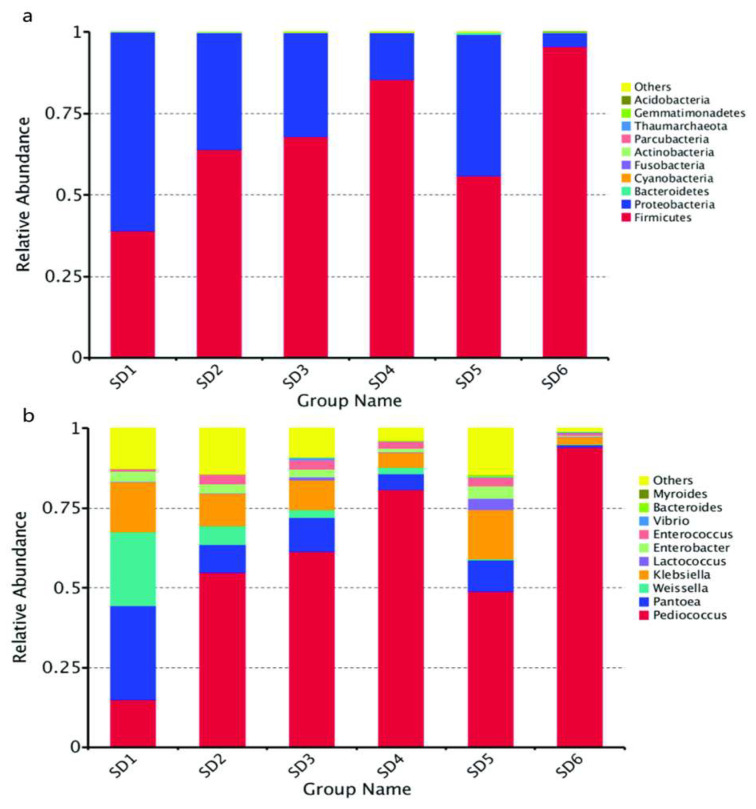
Relative abundance levels of bacterial phyla (**a**) and genera (**b**) during different fermentation stages of Huangjiu brewed from broomcorn millet.

The bacterial communities affect fermentation efficiency and play a decisive role in the flavor quality of Huangjiu. The bacterial community diversity increases during the initial fermentation stage [9]. The composition of bacterial communities found by high-throughput sequencing showed that 1,286,179 reads passed quality control, and 150 OTUs were obtained after clustering. According to the database comparison, we found 2 kingdoms, 11 phyla, 23 classes, 46 orders, 65 families, and 82 genera during broomcorn millet Huangjiu fermentation. As shown in Figure 1, *Firmicutes* dominated at the phylum level, followed by *Proteobacteria*, with the opposite trend to *Firmicutes*. *Pediococcus*, *Pantoea*, and *Weissella* were the dominant genera, with over 3/5 peaking on the 5th day. Both *Firmicutes* and *Pediococcus* increased gradually in the first 4 days of fermentation, with a decrease on the 5th day and reaching the highest level on the 6th day. *Proteobacteria*, *Pantoea*, and *Klebsiella* showed a decreasing trend and then increased on the 5th day.

#### 3.3.2. Bacterial Community Network Analysis during Fermentation

**Figure 2:**
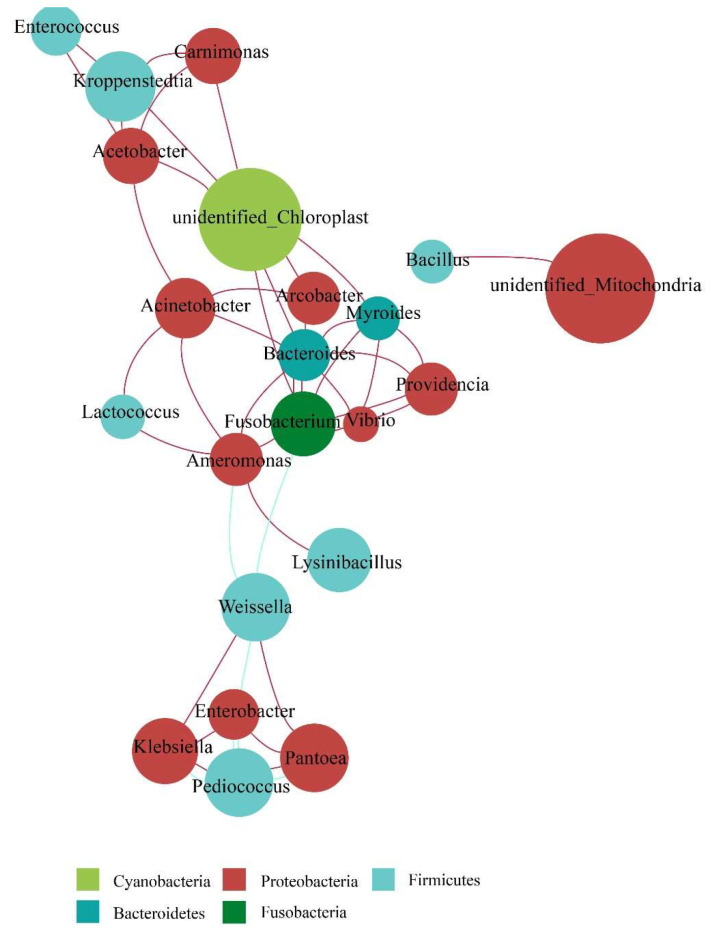
Bacteria correlation network analysis of Huangjiu brewed from broomcorn millet.

It was shown that *Bacteroides* established the most positive correlation with some genera, such as *Myroides*, *Arcobacter*, *Acinetobacter*, *Fusobacterium*, and *Providencia*. *Bacillus* spp. were rarely correlated with the other genera. Among LAB, *Lactococcus* was only positively correlated with Acinetobacter and *Ameromonas*. *Pediococcus* and *Weissella* were both positively associated with *Pantoea* and *Klebsiella* and negatively correlated with *Enterobacter* (Figure 2).

### 3.4. Fungal Diversity of Broomcorn Millet Huangjiu during Primary Fermentation

#### 3.4.1. Analysis of Fungal Community Dynamics

A total of 159,909 sequences were subjected to quality control filtering, and 143 OTUs were obtained after clustering. A total of 5 phyla, 11 classes, 20 orders, 29 families, and 30 genera were identified in this study. Ascomycota and Zygomycota were the dominant phyla. At the genus level, the fungal community was mainly dominated by *Saccharomyces* and *Rhizopus*, the abundance of Ascomycota had the greatest effect on differential by linear discriminant analysis (LDA), and Candida established the most associations by network analysis.

**Figure 3:**
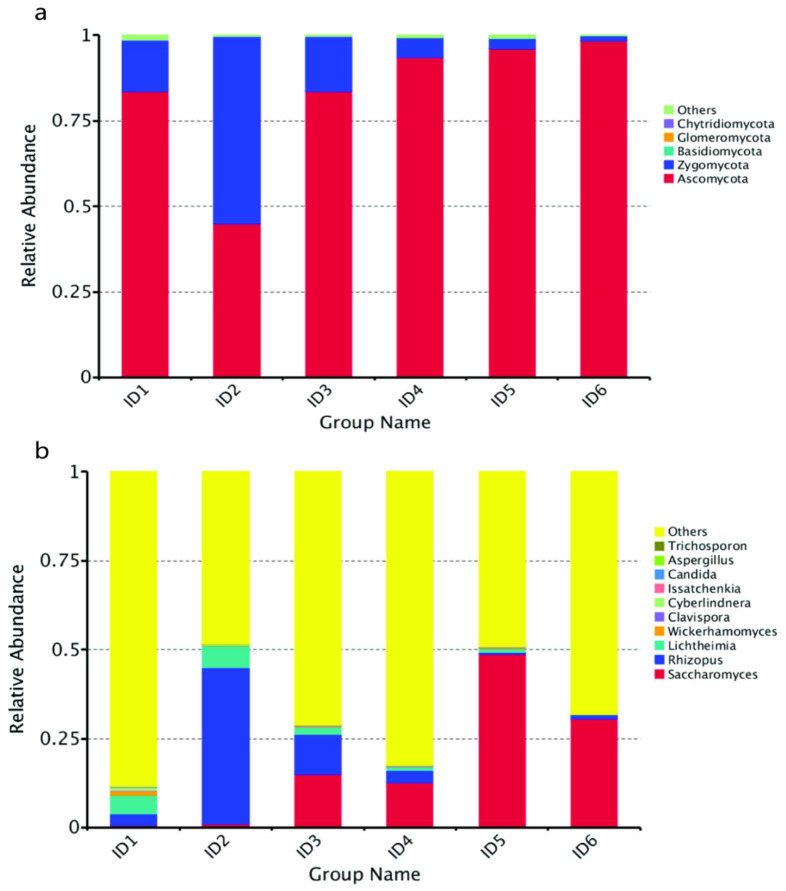
Relative abundance levels of fungi phyla (**a**) and genera (**b**) during different fermentation stages of Huangjiu brewed from broomcorn millet.

Approximately 96% of the ITS region sequences were assigned to *Ascomycota* and *Zygomycota*. Except for on the 2nd day, the dominant phylum was Ascomycota during the fermentation process (Figure 3a). *Rhizopus* peaked at the genus level on day 2, reached the highest level on the 8th day, then decreased rapidly until the end of fermentation. The *Saccharomyces* content was lower during the first 2 days of fermentation but dominated from the 3rd day until the end of fermentation. Compared to *Saccharomyces*, *Lichtheimia* showed the opposite tendency (Figure 3b).

#### 3.4.2. Fungal Community Network Analysis during Fermentation

**Figure 4:**
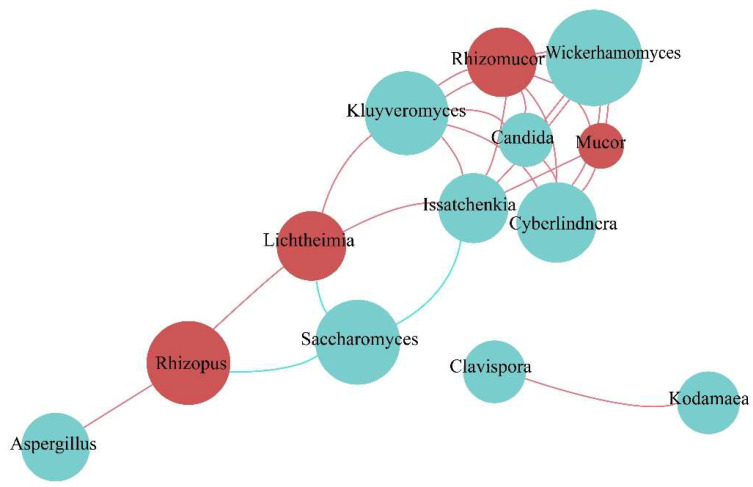
Fungi correlation network analysis of Huangjiu brewed from broomcorn millet.

As shown in Figure 4, *Candida* established the most positive relationships with other genera, such as *Rhizomucor*, *Wickerhamomyces*, *Mucor*, and *Issatchenkia,* during broomcorn millet Huangjiu fermentation. *Saccharomyces* showed a negative correlation with *Issatchenkia*, *Lichtheimia*, and *Rhizopus*, whereas *Aspergillus* was positively correlated with *Rhizopus*.

### 3.5. Correlation between Microorganisms and Volatile Flavor Compounds during Primary Fermentation

**Figure 5:**
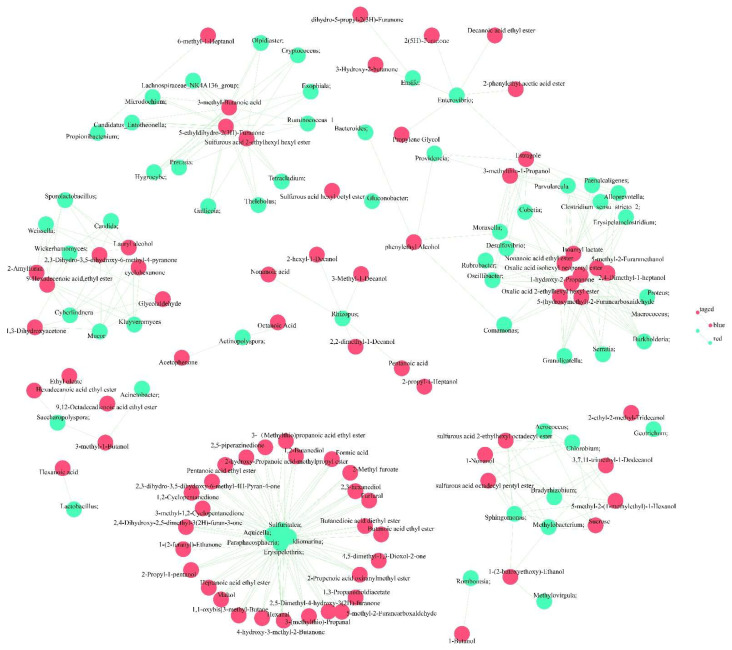
Correlation between microorganisms and flavor compounds in broomcorn millet.

To reveal the role of microbes in the formation of volatile flavor compounds during broomcorn millet Huangjiu fermentation, we examined the correlation between bacterial and fungal communities and volatile flavor compounds (*p* < 0.05). The results showed that a total of 1217 associations were established, involving 102 microorganisms and 101 flavoring substances (Figure 5). Among them, *Lactobacillus*, *Photobacterium*, *Hyphodontia*, *Aquicella*, *Erysipelothrix*, *Idiomarina*, *Paraphaeosphaeria*, and *Sulfuritalea* showed over 30 associations with volatile flavor compounds. Conversely, *Pediococcus* was found to be related only to ethyl myristate. Among the flavoring substances, 5-methyl-2-(1-methylethyl)-1-hexanol, the most complex flavoring substance in terms of association, was associated with 54 microorganisms. It was also shown that over 30 associations were established including nonanoic acid ethyl ester, 1-hydroxy-2-propanone, 2,4-dimethyl-1-heptanol, 5-(hydroxymethyl)-2-furancarboxaldehyde, isoamyl lactate, oxalic acid 2-ethylhexyl hexyl ester, oxalic acid isohexyl neopentyl ester, 2(5H)-furanone, 2-phenylethyl acetic acid ester, 3-methyl-1-butanol, 3-methylthio-1-Propanol, decanoic acid ethyl ester, and estragole.

### 3.6. Effects of the Quality of Broomcorn Millet Huangjiu with Fortified Qu Fermentation

#### 3.6.1. Effects of Biogenic Amine Content in Huangjiu

**Figure 6:**
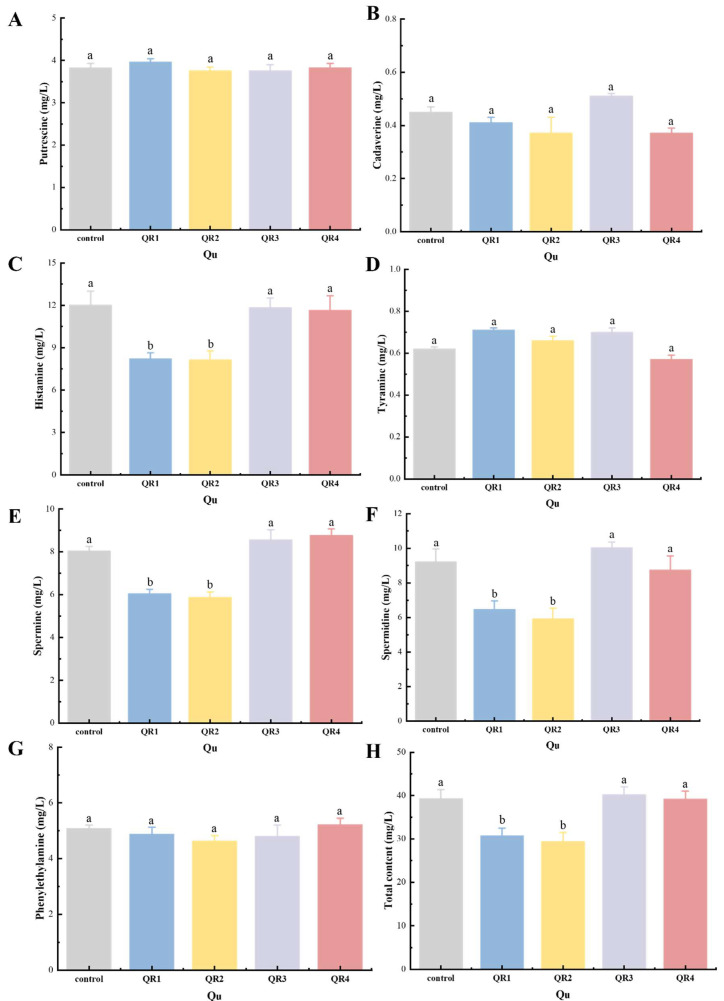
Comparison of bioamine content in wheat qu (control) and fortified qu (QR1, QR2, QR3, and QR4) fermentation. (**A**) Putrescine, (**B**) Cadaverine, (**C**) Histamine, (**D**) Tyramine, (**E**) Spermine, (**F**) Spermidine, (**G**) Phenylethylamine, (**H**) Total content. Different lowercase letters indicate significant differences in different groups (*p* < 0.05).

Four bacteria, *Lactobacillus* (R1), *Photobacterium* (R2), *Idiomarina* (R3), and *Pediococcus* (R4), were isolated and identified from wheat Qu, which were added to wine Qu to prepare four kinds of fortified Qu. The biogenic amine level was determined by HPLC. As shown in Figure 6, compared with the control group, there were no significant changes in the total biogenic amine content of Huangjiu brewed with QR3 and QR4. At the same time, the biogenic amine content of QR1 and QR2 was reduced by 21.9% and 22.2%, respectively, while the histamine, spermidine, and spermidine showed a lower content (*p* < 0.05). This indicates that adding R1 and R2 may significantly reduce the amount of biogenic amine in Huangjiu. This may be because the new bacteria R1 and R2 could effectively inhibit the growth and reproduction of amine-producing miscellaneous bacteria, resulting in further reduction of biogenic amines and promotion of the quality of Huangjiu.

#### 3.6.2. Analysis of Volatile Flavor Compounds in Huangjiu

To further determine the quality of Huangjiu, alkanes, alcohols, esters, ketones, aldehydes, acids, pyrazines, and phenols were detected. Among the groups, there were no significant differences in the total flavor substance content of broomcorn millet Huangjiu with QR3 and QR4. The content of aromatic compounds in the QR1 group increased by 16.1%, with a significant improvement in alcohols, esters, acids, and other classes of aromatic substances (*p* < 0.05). The total flavor content of QR2 also increased by 18.0%, among which alkanes, alcohols, esters, acids, and other flavor substances were observed to be higher than the control group (*p* < 0.05) (Table 2). Therefore, studies have shown that R1 and R2 fortified qu could significantly improve the flavor quality of Huangjiu.

#### 3.6.3. Analysis of Physicochemical Indexes in Huangjiu

The physicochemical index content of fermented Huangjiu is shown in Table 3. Huangjiu brewed with QR1, QR2, QR3, and QR4 showed no significant changes in alcohol, total sugars, and non-sugar solids compared with the control group, while QR1 and QR2 obviously increased amino acid nitrogen and total acid. Fortified qua (QR1 and QR2) may have affected the micro-ecosystem of Huangjiu, inhibited the growth and reproduction of bioamine-producing bacteria, reduced the conversion of amino acids into biogenic amines, and increased the accumulation of amino acids.

## 4. Discussion

Microorganisms have a complex symbiotic relationship during Huangjiu fermentation, thus, forming a unique flavor [13]. The use of wheat Qu is a feature of Huangjiu. Qu-derived microorganisms and environmental microorganisms are major microbial sources in the fermentation process, increasing the diversity and complexity of Huangjiu’s flavor. Due to the interaction between microorganisms during fermentation, different aromatic compounds are produced in Huangjiu [28]. Because of the sufficiency of nutrients and a favorable environment during the initial fermentation, fungal microorganisms grow rapidly and can produce many enzymes involved in cellular metabolism and the resultant small molecules, contributing to the formation of esters [29,30]. In this study, we found that *Pediococcus*, *Pantoea*, *Weissella*, *Rhizopus*, *Saccharomyces*, and *Lichtheimia* were the principal microorganisms produced during the fermentation of broomcorn millet Huangjiu. *Pediococcus* spp. are commonly used in silages, cheese, and yogurt [31,32]. Except for in some special flavored beers, *Pediococcus* is regarded as a contaminant in brewed beer and wine. Some *Pediococcus* species can produce diacetyl, which gives a buttery or creamy flavor to wine and beer [9]. *Pediococcus* has been reported to assist in German boche fermentation, which yields acidity to improve quality but has never been involved in Huangjiu fermentation [9]. Correlation analysis was performed to identify the specific correlations between microorganisms and volatile flavor compounds during fermentation. We found that *Pediococcus* spp. was strongly associated with ethyl myristate, while *Pantoea* showed a positive correlation with ethyl myristate content. Consequently, *Pantoea* and *Pediococcus* have a strong coordination effect during broomcorn millet Huangjiu fermentation. A positive correlation between *Weissella* and ethyl butyrate has been reported in traditional Chinese Xiaoqu rice wine [33]. *Rhizopus*, with its high amylase capacity, is closely bound to many organic acids such as pentanoic acid, nonanoic acid, and octanoic acid. Most species of *Saccharomyces* ferment one or more of the following sugars: galactose, xylose, maltose, disaccharide, trehalose, and sucrose. *Saccharomyces* was the only genus of fermented soluble starch, and it was closely associated with alcohols and esters such as propylene glycol and 2-phenylethyl acetic acid ester.

Aromatic esters and higher alcohols are regarded as two of the most important flavor characteristics of Huangjiu. Alcohols are ester precursors, which can enhance the sweetness of Huangjiu [5]. Esters are one of the most important volatile flavor components, with fruity and floral flavors in Huangjiu [5]. The alcohols present in Huangjiu include 2,3-butanediol, glycerol, 2-phenylethanol, 2-methyl-1-propanol, and 3-methyl-1-butanol. Most of these alcohols are degradation products of amino acids [34]. These strains are modified by synthetic biology or the application of genetic engineering techniques that can produce 2-methyl-1-propanol [35,36]. Based on the expression of *kivd* and *adh2* genes in *Escherichia coli*, Atsumi et al. showed that the isobutanol is biosynthesized via the Ehrlich pathway using 2-ketoisovaleric acid, an intermediate of amino acid synthesis pathway, as a raw material [35]. The association analysis in this study concluded that 2-methyl-1-propanol is associated with Halomonas. The physiological, metabolic pathway of Halomonas has not been reported, so the association with 2-methyl-1-propanol needs to be verified in the future. 3-methyl-1-butanol was the main hybrid oleyl alcohol component. The higher 3-methyl-1-Butanol should be controlled during fermentation because excessive concentration affects the quality of the wine; subsequent studies have shown that isoamyl alcohol is an important bitter compound in Shaoxing Huangjiu [37]. Our results indicated that *Rhizopus*, *Aspergillus*, and over 30 genera of microorganisms are associated with 3-methyl-1-Butanol production. However, whether 3-methyl-1-butanol content can be regulated by these genera has not yet been fully investigated. 2, 3-butanediol, a warm, sweet flavor compound, is usually added to white wine in China to improve its flavor. Some bacteria, such as *Klebsiella oxytoca*, show a high potential for 2, 3-butanediol production [38]. *Pantoea* and *Kluyveromyces* are associated with the synthesis of 2, 3-butanediol during the brewing of broomcorn millet Huangjiu. Furthermore, aromatic esters are broadly produced via the combination of organic acids and alcohols during fermentation, amino acids with alcohols, and decomposition, combination, and oxidation reactions during the aging of Huangjiu [39]. The 32 esters build associations with 92 microorganisms. Among these, *Idiomarina* can produce amylase, esterase, trypsin, and other fermentation-friendly enzymes. *Fusobacterium* usually produces a mixture of organic acids and alcohols using sugars or peptones. *Ochrobactrum* uses amino acids, organic acids, and carbohydrates as carbon sources.

In addition to aromatic esters and higher alcohols, a momentous microbial metabolite, Acetoin (3-hydroxy-2-butanone), was reported as an important quality indicator and flavor substance for fermented products by a previous study [40]. In our study, acetoin formation was related to 28 different genera during millet Huangjiu primary fermentation, indicating that acetoin biosynthesis is a complex process that involves many microorganisms in addition to the reported genus. Estragole is a colorless or light-yellow liquid with a strong aniseed aroma and sweetness, which are important fragrances, bioactive substances, and active intermediate [41]. Estragole synthesis was associated with 31 microorganisms, such as *Moraxella*, *Enterovibrio*, *Lactococcus*, and *Pseudomonas,* according to our study; furthermore, this required further analysis of the interactions between them.

R1, R2, and R3 were most associated with Huangjiu flavor substances, and R4 was the dominant bacterium. Compared with the wheat qu, there was no significant difference between Huangjiu brewed with QR3 and QR4, while using QR1 and QR2 showed significantly lower biogenic amine content, higher flavor substance, and amino acid nitrogen content, which improved the quality of Huangjiu.

## 5. Conclusions

In this study, HS-SPME/GC-MS was used to determine 161 volatile flavor compounds, mainly esters, alcohols, acids, aldehydes, ketones, and phenols. A total of 82 and 30 genera of bacteria and fungi, respectively, were detected using high-throughput sequencing. We found 1217 correlations between microbes and flavor compounds. The results indicated that the dominant genera of flavor compounds were *Lactobacillus*, *Photobacterium*, *Hyphodontia*, *Aquicella*, *Erysipelothrix*, *Idiomarina*, *Paraphaeosphaeria*, and *Sulfuritalea*. 5-methyl-2-(1-methylethyl)-1-Hexanol is the most complex flavoring substance and is associated with 54 microorganisms. Estragole was first detected in broomcorn millet Huangjiu. Furthermore, the addition of QR1 and QR2 could improve Huangjiu’s quality by increasing flavoring substances, the amino acid nitrogen content, and acidity while decreasing the bioamine content. This work reveals the relationship between microorganisms and volatile flavor compounds and is beneficial for the regulation of the micro-ecosystem and flavor of broomcorn millet Huangjiu.

## Figures and Tables

**Table 1:** Changes in acid, reducing sugar, and alcohol found in Huangjiu brewed from broomcorn millet during primary fermentation.

Compounds (g/L)	Fermentation Time (Days)					
	1st	2nd	3rd	4th	5th	6th
acid	1.71 ± 0.07	2.43 ± 0.07	2.71 ± 0.04	4.90 ± 0.15	5.32 ± 0.11	6.41 ± 0.16
reducing sugar	27.65 ± 0.97	32.35 ± 0.88	26.55 ± 0.91	24.25 ± 1.09	22.45 ± 0.89	19.14 ± 0.62
alcohol	2.32 ± 0.13	10.62 ± 1.35	18.54 ± 1.27	29.83 ± 1.45	53.16 ± 2.19	61.33 ± 3.25

All values are expressed as mean standard error (*n* = 3).

**Table 2:** Comparison of volatile flavor compounds in wheat qu (control) and fortified qu (QR1, QR2, QR3, and QR4) fermentation.

Flavor Component	Control	QR1	QR2	QR3	QR4
Alkane	85.51 ± 3.67 a	90.25 ± 3.64 a	95.37 ± 5.85 b	88.43 ± 3.58 a	90.52 ± 3.61 a
Alcohol	151.25 ± 4.38 a	164.37 ± 4.83 b	166.23 ± 5.45 b	147.64 ± 5.09 a	152.85 ± 4.69 a
Ester	52.02 ± 3.11 a	70.35 ± 2.84 b	68.38 ± 3.72 b	54.07 ± 4.19 a	55.09 ± 3.24 a
Ketone	1.43 ± 0.02 a	1.71 ± 0.13 a	1.74 ± 0.04 a	1.60 ± 0.05 a	1.51 ± 0.04 a
Aldehyde	1.08 ± 0.01 a	0.92 ± 0.02 a	0.95 ± 0.01 a	0.86 ± 0.02 a	0.98 ± 0.02 a
Acid	2.34 ± 0.04 a	9.54 ± 0.27 b	11.25 ± 0.18 b	3.05 ± 0.08 a	2.19 ± 0.05 a
Pyrazine	0.03 ± 0.002 a	0.12 ± 0.003 a	0.06 ± 0.001 a	0.04 ± 0.002 a	0.03 ± 0.001 a
Phenol	8.05 ± 0.41 a	10.85 ± 0.74 a	9.54 ± 0.36 a	8.37 ± 0.42 a	8.86 ± 0.59 a
Others	9.04 ± 0.76 a	12.73 ± 1.86 b	13.21 ± 0.96 b	8.78 ± 0.33 a	9.43 ± 0.37 a
Total	310.75 ± 7.39 a	360.84 ± 5.36 b	366.73 ± 6.81 b	312.84 ± 6.26 a	321.19 ± 8.04 a

Control: wheat qu; QR1: qu fortified with R1; QR2: qu fortified with R2; QR3: qu fortified with R3; QR4: qu fortified with R4. Different lowercase letters in the same row indicate significant differences in different groups (*p* < 0.05).

**Table 3:** Comparison of physicochemical indexes in wheat qu (control) and fortified qu(QR1, QR2, QR3, and QR4) fermentation.

Indexes (g/L)	Control	QR1	QR2	QR3	QR4
Total acid	4.07 ± 0.61 a	6.82 ± 0.39 b	6.47 ± 0.39 b	4.32 ± 0.52 a	4.21 ± 0.54 a
Total sugar	77.28 ± 2.87 a	76.68 ± 3.19 a	76.51 ± 2.82 a	80.02 ± 2.92 a	76.38 ± 2.51 a
Non-sugar solid	19.36 ± 2.01 a	18.35 ± 2.14 a	18.06 ± 1.74 a	20.04 ± 1.87 a	20.04 ± 2.53 a
Alcohol	11.25 ± 1.34 a	12.06 ± 1.25 a	11.88 ± 1.54 a	10.93 ± 1.28 a	11.27 ± 1.45 a
Amino acid nitrogen	0.33 ± 0.02 a	0.52 ± 0.02 b	0.54 ± 0.01 b	0.35 ± 0.02 a	0.35 ± 0.02 a

Control: wheat qu; QR1: qu fortified with R1; QR2: qu fortified with R2; QR3: qu fortified with R3; QR4: qu fortified with R4. Different lowercase letters in the same row indicate significant differences in different groups (*p* < 0.05).

## Data Availability

The data presented in this study are available on request from the corresponding author.

## Supplementary Materials

The following supporting information can be downloaded at: https://www.mdpi.com/article/10.3390/foods12142680/s1. Table S1: Changes in volatile compounds in broomcorn millet Huangjiu during primary fermentation.
